# Clinical Review of Hypertensive Acute Heart Failure

**DOI:** 10.3390/medicina60010133

**Published:** 2024-01-10

**Authors:** Ratko Lasica, Lazar Djukanovic, Jovanka Vukmirovic, Marija Zdravkovic, Arsen Ristic, Milika Asanin, Dragan Simic

**Affiliations:** 1 Department of Cardiology, Emergency Center, University Clinical Center of Serbia, 11000 Belgrade, Serbia; drlasica@gmail.com (R.L.); lazardjukanovic08@gmail.com (L.D.); masanin2013@gmail.com (M.A.); 2 Faculty of Medicine, University of Belgrade, 11000 Belgrade, Serbia; sekcija.kardioloska@gmail.com (M.Z.); arsen.ristic@med.bg.ac.rs (A.R.); 3 Faculty of Organizational Sciences, University of Belgrade, 11000 Belgrade, Serbia; jovanka.vukmirovic@fon.bg.ac.rs; 4 Clinical Center Bezanijska Kosa, 11000 Belgrade, Serbia; 5 Department of Cardiology, University Clinical Center of Serbia, 11000 Belgrade, Serbia

**Keywords:** hypertension, acute heart failure, congestion, diagnosis, modern therapy

## Abstract

Although acute heart failure (AHF) is a common disease associated with significant symptoms, morbidity and mortality, the diagnosis, risk stratification and treatment of patients with hypertensive acute heart failure (H-AHF) still remain a challenge in modern medicine. Despite great progress in diagnostic and therapeutic modalities, this disease is still accompanied by a high rate of both in-hospital (from 3.8% to 11%) and one-year (from 20% to 36%) mortality. Considering the high rate of rehospitalization (22% to 30% in the first three months), the treatment of this disease represents a major financial blow to the health system of each country. This disease is characterized by heterogeneity in precipitating factors, clinical presentation, therapeutic modalities and prognosis. Since heart decompensation usually occurs quickly (within a few hours) in patients with H-AHF, establishing a rapid diagnosis is of vital importance. In addition to establishing the diagnosis of heart failure itself, it is necessary to see the underlying cause that led to it, especially if it is de novo heart failure. Given that hypertension is a precipitating factor of AHF and in up to 11% of AHF patients, strict control of arterial blood pressure is necessary until target values are reached in order to prevent the occurrence of H-AHF, which is still accompanied by a high rate of both early and long-term mortality.

## 1. Introduction

Acute heart failure is defined as the rapid or gradual appearance of pronounced signs and/or symptoms of heart failure, which often require the patient to seek emergency medical help and which in most cases leads to unplanned hospitalization [1]. AHF does not include heart failure with moderate symptoms and signs, which can be treated on an outpatient basis by changing lifestyle habits and intensifying the medicinal therapy regimen.

Although the classification of heart failure has changed over the years, it is clearly accepted that the classification of chronic heart failure is based on the left ventricular ejection fraction (LVEF) value [2]. So far, different divisions of AHF have been attempted with the aim of clearly defining this entity to ensure easier application of an adequate therapeutic regimen for each phenotype. The initial opinion that every acute heart failure is a consequence of fluid volume overload was rejected by analyzing the pathophysiological mechanisms involved in its occurrence. The term H-AHF contains both a clinical and a pathophysiological component. Although it has been shown that close to 50% of patients with AHF are hypertensive, not all of them belong to H-AHF. The primary precipitating pathophysiological factor in the development of H-AHF is vasoconstriction, which leads to pulmonary edema as a manifestation of the sudden onset of H-AHF [2]. The term hypertensive acute heart failure was first mentioned in the 2008 ESC recommendations for heart failure. Earlier recommendations for heart failure from 2001 and 2005 classified hypertension as an etiological factor of acute heart failure, but the term hypertensive acute heart failure itself is not mentioned. The 2008 guideline of the European Association of Cardiology for the treatment of heart failure proposed six different clinical forms of AHF: worsening or decompensated chronic HF; pulmonary edema; hypertensive HF; cardiogenic shock; isolated right HF; and HF in acute coronary syndrome [3]. In the same year, a modification of the AHF categories derived from the Forrester classification for heart failure after myocardial infarction was proposed, which is based on the presence or absence of tissue congestion and perfusion [4]. The heart failure guide from 2012 proposed the classification of AHF based on the level of systolic blood pressure (SBP) at the initial presentation of the patient [5,6]. All of the aforementioned divisions of AHF were analyzed within the ESC Heart Failure Long-Term (HF-LT) registry and the study published by Chioncel et al. In that study were analyzed 6629 hospitalized patients with AHF, and it was shown that there are significant differences in early mortality and adverse events depending on the clinical profile of the patient or the value of arterial blood pressure at the initial examination. This difference was especially registered in patients in the first 6 months of follow-up, while after that time period, the one-year outcome of the patient was less influenced by the clinical profile or SBP value at admission [6]. According to the 2016 ESC guide, AHF classification was mainly based on phenotypes resulting from the combined ratio of congestion and hypoperfusion (wet and dry, cold and warm) [7]. By combining them, four different phenotypes were created. In the study by Javaloyes P. and associates, this division was shown to be very simple in clinical practice, and the authors emphasized the importance of clinical assessment of congestion and perfusion at the initial presentation right next to the patient’s bed. According to their data, the clinical classification into four phenotypic profiles correlated with the early outcome of these patients and was helpful to doctors in making a more precise decision when administering a therapeutic regimen to patients with AHF [2,8]. Other studies conducted on large registries of patients with heart failure showed similar results [4,9,10]. On the other hand, Masip J. and colleagues also mentioned the negative aspects of this division of AHF. The incidence of the mentioned phenotypes is very unbalanced; patients belonging to the “cold and dry” group occurred with a frequency of less than 1%, while patients with the “wet and warm” phenotype made up about 80% of treated patients. Also, with the concept of congestion, no distinction was made between systemic and pulmonary congestion. The third remark according to those authors is that the patient cannot have AHF that requires urgent hospital treatment without congestion or hypoperfusion, so the “warm and dry” phenotype is debatable [11]. In 2016, the Consensus Statement from the Society of Academic Emergency Medicine and the Heart Failure Society of America Acute Heart Failure Working Group was published, which deals exclusively with the pathology of hypertensive acute heart failure. In accordance with the current knowledge, the latest division from 2021 focused to the greatest extent on the pathophysiological mechanisms that lead to AHF. According to this classification, AHF is divided into acute decompensated heart failure, acute pulmonary edema, isolated right ventricular failure and cardiogenic shock [1].

Despite all efforts to find the best clinical classification of AHF that would make it easier for doctors to choose an individual therapeutic regimen for each patient, mortality from AHF is still very high [12]. We authors think that the main goal is to see the pathophysiological changes in AHF that are responsible for the emergence of difficult clinical scenarios. Patients with H-AHF present a clinical phenotype dominated by an increase in afterload and a decrease in venous capacitance as well as a consequent increase in ventricular filling pressures. In this paper, we will consider the pathophysiological mechanisms underlying H-AHF as well as the current data on the best options for its treatment.

## 2. Materials and Methods

In the preparation of this paper, an electronic search was performed on reliable databases (PubMed, Web of Science, Scopus, Google Scholar) in order to identify all relevant reports on H-AHF. A comprehensive search strategy was developed by the authors based on the following keywords: hypertensive acute heart failure; hypertension; acute heart failure; pulmonary edema.

## 3. Epidemiology

Most epidemiological data related to AHF are obtained from large heart failure registries [13,14,15,16,17,18]. In different registries, the frequency of certain phenotypic forms of AHF is different. According to the ALARM-HF registry, the frequency of acutely decompensated congestive HF was 38.6%, pulmonary edema 36.7% and cardiogenic shock 11.7%. Cardiogenic shock was less prevalent according to other registries, with about 2–5% [6,19]. In analyzing the results of the registers, it was shown that the largest number of patients with AHF already have known heart failure (about two-thirds of patients), while the number of patients with de novo AHF is much smaller. About 50% of patients with already known chronic heart failure have preserved EF. The gender ratio is mostly symmetrical with a slight male predisposition. Patients with AHF have hypertension in about 70% [14,16]. Arterial hypertension is more common in HF patients with preserved LVEF (76%) compared to those with reduced LVEF (66%) [20]. Data from the STAT register, which monitors patients hospitalized for hypertensive crisis, indicate that about 25.2% of patients with hypertensive crisis have AHF. Chiocel O. et al. point out that H-AHF constitutes 4.8% of all forms of AHF [6]. However, other authors point out that the share of H-AHF within the total AHF is much higher, up to 11% [21].

## 4. Pathophysiological Mechanisms of H-AHF

Hypertensive acute heart failure is defined as the rapid onset of pulmonary congestion in the setting of a systolic blood pressure >140 mmHg and often >160 mmHg [22]. Most patients with H-AHF have previously known heart failure (usually with preserved EF) and long-standing hypertension [20,23]. Maintaining blood pressure is strictly regulated by means of baroreceptors, primarily in the aorta and carotid arteries [24]. A significant role in maintaining normal values of arterial blood pressure is also played by renal regulation mechanisms (the influence of the renin–angiotensin–aldosterone system in the regulation of arterial blood pressure, affecting the volume of circulating blood as well as vascular resistance and tone). The influence of renal mechanisms is reflected in the fact that approximately 20–30% of patients with HF have some degree of renal weakness [25].

Changes in cardiac output and systemic vascular resistance trigger sympathetic nervous system and neurohumoral activation [26]. A heart with normal contractility is able to respond promptly to an increase in systemic arterial resistance and to maintain an adequate cardiac output. When a pressure load occurs, the left ventricle undergoes hypertrophic structural remodeling [27]. As the end result of these changes, there is a slow relaxation of the left ventricle with significant diastolic dysfunction. As the functional ventricular–vascular relationship becomes uncoupled, the LV has insufficient cardiac reserve to compensate for the increases in afterload and preload that accompany hypertensive episodes and physical exertion [24,28]. As a result of all this, the cardiac output is unable to increase in response to increased systemic vascular resistance, which leads to increased volume and pressure in the left ventricle and impaired blood flow from the pulmonary veins to the heart, which predisposes the patient to pulmonary congestion. Such increases in filling volume also trigger the Frank–Starling mechanism in the right ventricle (RV), which combines a catecholamine-mediated increase in RV contractile force to drive up pulmonary artery and capillary wedge pressures [24]. The end result of all these processes is the appearance of pulmonary congestion. According to the study by Chiolance J. and colleagues, within the framework of the H-AHF phenotype, the largest number of patients present with pulmonary congestion—as many as 66% [6]. About 10% of patients have peripheral hypoperfusion with varying degrees of pulmonary congestion, while hypoperfusion without pulmonary congestion is rarely present among H-AHF patients. In one-half of all patients with AHF, several different precipitating factors are involved that lead to decompensation in AHF patients [29].

**Figure 1:**
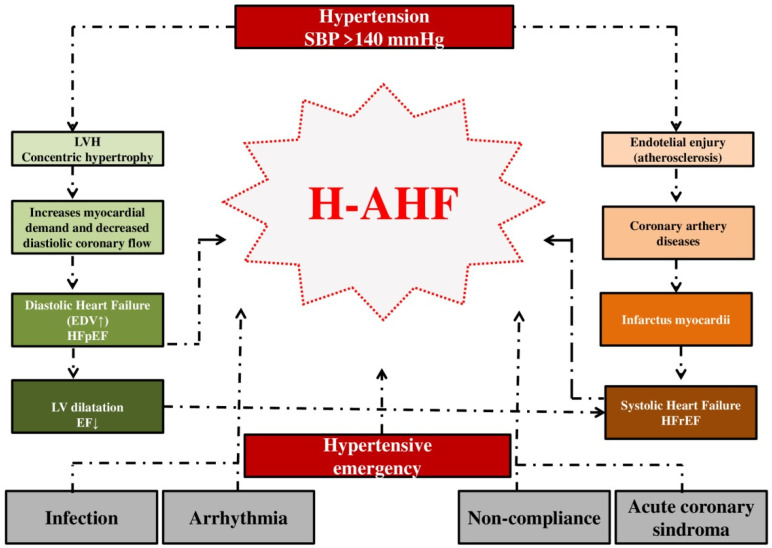
**The influence of hypertension on the development of hypertensive acute heart failure. Legend: SBP** = systolic blood pressure; **LV** = left ventricular; **LVH** = left ventricular hypertrophy; **EDV** = end-diastolic volume; **EF** = ejection fraction; **HFpEF** = heart failure with preserved ejection fraction; **HFrER** = heart failure with reduced ejection fraction; **H-AHF** = hypertensive acute heart failure.

In patients with arterial hypertension, maladaptive changes occur in the myocardium and vasculature as a response to chronic hypertension, creating a system extremely sensitive to changes in pressure, fluid volume and sympathetic tone. Peripheral and central baroreceptors become tolerant to higher pressures; in fact, the aortic baroreflex becomes blunted [27]. Structural disorders in peripheral arterioles and central arteries functionally separate the vascular system from the ventricular system in hypertensive patients [30,31]. The primary pathophysiology in hypertensive AHF centers on a mismatch in the ventricular–vascular coupling relationship. Apart from the role in the transport of blood to the organs, the importance of blood vessels is also in mitigating the maximum systolic blood pressure and maintaining nonpulsatile diastolic flow. Primarily, hypertension can affect an increased stiffness of blood vessels, decrease in their compliance and increased resistance to flow from the left ventricle [24,32]. As a result of the decrease in compliance, there is an increase in the vascular resistance of the peripheral blood vessels, while the end-diastolic pressure increases in the heart chamber. Peripheral and splanchnic vasoconstriction, in addition to increasing afterload, also lead to a redistribution of blood and increase in preload [33]. With the increase in pressure and volume in the chamber, there is retrograde expansion of the pulmonary circulation and the consequent appearance of congestion [27]. However, to a certain extent, even with this AHF phenotype, fluid volume retention can be an additional significant factor that leads to acute HF [34] (Figure 1).

## 5. Clinical Picture of H-AHF

In patients with H-AHF, cardiac decompensation usually occurs quickly, within a few hours to a few days [21,35,36]. Often the only precipitating factor for AHF is arterial hypertension. Patients usually do not complain of weight gain, nor do they have swelling on the lower legs that occurs due to peripheral congestion. At first examination, they have severely elevated SBP (≥160–180 mmHg) with auscultatory signs of pulmonary congestion [21]. The clinical picture is dominated by dyspnea. The patient takes a sitting position because it is easier to breathe.

The most extreme presentation of H-AHF is pulmonary edema. Clinical criteria for acute pulmonary edema diagnosis include dyspnea with orthopnea, respiratory failure, tachypnea (>25 breaths/min) and increased work of breathing [37]. Accumulation of fluid in the lungs leads to impaired gas exchange and arterial hypoxemia. In patients with pulmonary congestion, juxtapulmonary capillary (J-type) receptors are stimulated, which leads to tachypnea [38]. Thus, most AHF patients with pulmonary edema hyperventilate due to J-type receptor stimulation before a significant pathologic change in gas exchange occurs [38]. Since CO_2_ has a better diffusing capacity than O_2_, CO_2_ is less likely to increase until the later stages of diaphragmatic fatigue [38,39,40]. Symptoms that correlate with elevated arterial blood pressure are often present: headache, visual disturbances, chest pain, dizziness [41]. Patients may also have some neurological deficits [42].

Patients with H-AHF may have central cyanosis. On auscultation over the lungs, the presence of a weakened respiratory sound on both sides may be a consequence of the presence of pleural effusions, while the finding of inspiratory cracks is in favor of fluid transudation into the pulmonary alveoli [43,44]. Auscultation of the heart often registers the presence of a third heart sound.

Although patients presenting with H-AHF appear the sickest and are assessed as high-risk by physicians, they actually have the most favorable clinical outcome and prognosis of all AHF phenotypes [22,45,46,47]. The estimated in-hospital mortality of these patients is about 1.8% [6].

## 6. Laboratory and Multimodal Imaging in the Diagnosis of H-AHF

In patients with AHF, in addition to establishing the diagnosis of HF itself, it is necessary to see if the cause is de novo heart failure or to detect all potential precipitating factors and influence them. Physical examination is a fundamental component in the evaluation, risk stratification and outcome prediction of patients with AHF [48]. Patients with physical examination findings consistent with volume overload (such as elevated jugular venous pressure and presence of peripheral edema) have higher body mass index, higher biomarker values, more precipitating factors for AHF and lower LVEF compared to those without these findings.

Electrocardiography is a method that does not have sufficient specificity or sensitivity to diagnose AHF. It is very important in diagnosing various rhythm disorders that can be both a triggering factor for the appearance of AHF and a consequence of it. Its greatest importance is reflected in the exclusion of ischemic causes of AHF [49,50]. In addition to the ECG, the measurement of cardiospecific enzymes such as troponin can serve this purpose. Studies showed that troponin values can be moderately elevated in patients with acute heart failure, and positive troponin values have prognostic significance for the outcome of patients with AHF [51,52]. Analysis of data from the ADHERE observational registry for heart failure showed that elevated TnT values at admission in patients with heart failure are correlated with lower LVEF. Patients with positive TnT values have a higher in-hospital mortality compared to those patients who had negative TnT values (8% vs. 2.7%), with an adjusted OR of 2.55 for risk of death [53]. Similar data were shown by the EFECT study conducted on patients with AHF. A peak cTnI >0.5 ug/L measured in the first 48 h of hospitalization was an independent predictor of all-cause mortality at 1 year with an HR of 1.49 [54]. In AHF that is accompanied or caused by a hypertensive crisis, elevated troponin values are registered [55]. In order to rule out ischemic causes of AHF, it is necessary to correlate the patient’s clinical picture, changes in the ECG and dynamics in troponin values.

The main stimulus for increased brain natriuretic peptides (BNP) and N terminal pro brain natriuretic peptide (NT-proBNP) synthesis and secretion is myocardial wall stress. The physiological effects of BNP are manifold and include natriuresis/diuresis, peripheral vasodilatation and inhibition of the renin–angiotensin–aldosterone system and the sympathetic nervous system [56]. In a large number of studies, BNP and NT-proBNP were consistently found to be elevated in patients with AHF, and the values were found to be related to the severity of the disease (they are higher in patients with a more severe clinical picture, lower LVEF and a more severe degree of diastolic dysfunction) [57,58,59]. During the evaluation of patients with acute dyspnea, it is recommended to measure the value of natriuretic peptides [60]. According to the PRIDE study, NT-proBNP measurement is a valuable addition to standard clinical assessment for the identification and exclusion of AHF in the emergency department setting [61]. Studies have also shown the importance of negative BNP values for excluding AHF in patients who presented with dyspnea in the emergency department [62]. In general, HF is unlikely at BNP values < 100 pg/mL and is very likely at BNP values > 500 pg/mL and, similarly, unlikely at NT-proBNP values < 300 pg/mL and very likely at NT-proBNP values > 450 pg/mL (>900 pg/mL in patients over 50 years of age) [56]. It is important to note that natriuretic peptides can be elevated both in AHF and in patients with chronic HF, so the measurement of natriuretic peptides is more important in distinguishing between causes of cardiac and noncardiac origin of AHF than in distinguishing between AHF and chronic HF [63]. Low concentrations of BNP can sometimes be registered in patients with advanced decompensated end-stage HF, in obese patients and in patients with flash pulmonary edema or right-sided AHF [1]. A more recent study by Dal Bianco J.P. and colleagues, who examined BNP values in patients with flash edema and different degrees of cardiac function, showed that BNP levels were elevated in every patient, even when BNP was assayed early after dyspnea onset [64]. In addition to BNP, studies demonstrated that soluble suppressor of tumorigenicity 2 (sST2), GDF-15, cystatin C, galectin-3, serum uric acid, microRNAs and low serum chloride are predictors of outcomes in AHF [65,66,67]. In patients with cardiorenal syndrome, it is important to monitor markers related to renal function. Deterioration of renal function accompanied by a rise in plasma urea and creatinine is associated with a risk of increased mortality and new hospitalizations [68,69]. Proenkephalin levels, a novel marker of renal function, are also associated with worsening renal function and in-hospital and follow-up mortality in patients with AHF [70].

Transthoracic echocardiography should be performed in all de novo AHF or in patients with decompensated chronic HF when a cardiac pathology is suspected in order to evaluate the function of the left and right ventricles, the presence of segmental outbursts in the kinetics of the left ventricle, valve function and the presence of fluid in the pericardium [71]. All patients who come to the emergency department due to dyspnea must undergo teleradiography of the heart and lungs [72]. Its importance in the differential diagnosis of various lung diseases has been clearly demonstrated [73]. On the other hand, the presence of congestion on the X-ray of the heart and lungs can largely confirm the suspicion that it is AHF. Lung ultrasound proved to be a valid instrument to detect an increase in the superficial density and air space distribution of the lung [74]. Acute pulmonary edema of cardiogenic origin and acute respiratory distress syndrome (ARDS) are diseases that increase the density of the superficial lung and the full/empty ratio of the subpleural lung tissue, but in different ways [74,75]. Therefore, lung ultrasonography is generally considered a useful clinical tool among physicians. An inhomogeneous bilateral pattern of multiple coalescent B-lines and white lung, often with scattered spared areas, clearly characterizes ARDS, whereas the relatively regular presence of discrete B-lines characterizes the initial stages of pulmonary cardiogenic edema [76]. The criteria for the ultrasound differential diagnosis between ARDS and cardiogenic pulmonary edema were proposed in 2008 and revised in 2017 [76,77]. The importance of this technique is that it can be performed in a cheap, quick and simple way at the patient’s bedside.

Every patient with AHF should have arterial blood gas analyses. The most common gas exchange disorders in AHF are normoxemia or hypoxemia with hypocapnia [78]. The results of the study that examined gas exchange in arterial blood in patients with HF indicate that about 19% of patients with AHF had acidosis, 37% had normal pH and 44% had alkalosis. The most common type of acidosis was mixed-type (42%) followed by metabolic (40%), whereas the most common type of alkalosis was respiratory (58%). Acidosis proven in gas analyses was a significant predictor of mortality (hazard ratio 1.93; 95% confidence intervals 1.27–2.93) [38]. In contrast, alkalosis was not associated with increased mortality. In the study, 19% of the patients had acidosis and most patients had metabolic or mixed-type acidosis, whereas a pure respiratory acidosis was not common, suggesting that tissue hypoperfusion was the main cause of the acidosis, not CO_2_ retention as a result of impaired gas exchange [38].

In all patients with H-AHF, we must also determine inflammatory markers because only an infectious cause as a precipitating factor for AHF can be ruled out by measuring inflammatory markers (CRP, procalcitonin) [79].

## 7. Treatment of Patients with H-AHF

Treatment of acute heart failure remains a challenge for physicians. Recent data showed that timely initiation of therapy may be a key factor in the treatment of H-AHF [80]. Monitoring of vital parameters is necessary for all patients with AHF. Although the initial treatment should be started already during the diagnostic procedures, further evaluation of the started therapy should be directed both in relation to the clinical phenotype of AHF and to the potential causes that led to it. Acute coronary syndrome, acute hypertensive disorder, rapid ventricular response arrhythmias or severe bradycardia/conduction disorder, acute valvular regurgitation or infection (including myocarditis) should be considered [1]. Reducing ventricular filling pressure is a key component of management, especially when AHF is accompanied by hypertension [21]. Given that most patients with AHF have some degree of volume overload or present pulmonary/systemic congestion, diuretic therapy and vasodilator therapy still comprise the basic therapeutic regimen [24]. In patients with H-AHF, it is recommended to use vasodilators that optimize preload and afterload by decreasing venous and arterial tone and consequently lower SBP and increase stroke volume [81]. As the weakened heart is sensitive to afterload in some patients, pulmonary edema can occur even at SBP values of up to 150 mmHg [24].

The 2016 ESC guideline recommendations for the treatment of HF advise the treatment of AHF followed by a hypertensive crisis to rapidly reduce SBP (in the range of 25% during the first few hours and cautiously thereafter) [7]. It is necessary to consider the use of vasodilators in all patients with SBP values above 110 mmHg. Kitai T. et al. analyzed data from the REALITY-AHF registry in which AHF patients were analyzed [81]. Patients who received vasodilator therapy with consequent SBP reduction ≤ 25%, vasodilator therapy with >25% SBP reduction and no vasodilators (mean arrival SBP 149 ± 37 mmHg) were compared. Patients treated with vasodilators and with ≤ 25% SBP reduction at 6 h after initial presentation had a greater diuretic response and lower 1-year mortality (HR 0.74; 95%CI 0.57–0.96) compared to patients with ≥ 25% reduction or no vasodilator.

### 7.1. Vasodilators

#### 7.1.1. Nitrates

Nitrates are primarily venodilators, and their application leads to a decrease in venous inflow to the heart, which reduces the possibility of congestion, lowers afterload and increases stroke volume and consequent relief of symptoms. The use of nitroglycerin in the treatment of H-AHF is accompanied by a rapid onset of action of the drug, and the reduction in the overload and afterload depends on the applied dose [24]. Nitrates are generally administered with an initial bolus followed by continuous infusion. Nitroglycerin can be given as a 1–2 mg bolus in severely hypertensive patients with acute pulmonary edema [1]. Levy et al. reported aggressive BP control with very-high-dose nitroglycerin was associated with fewer intensive care unit (ICU) admissions and less endotracheal intubation compared to historical controls [82]. The Vasodilation in the Management of Acute CHF (VMAC) trial failed to demonstrate statistically significant improvement of pulmonary capillary wedge pressure or self-reported dyspnea scores 3 h after the initiation of nitroglycerin infusion compared with placebo [83]. It is significant that a significantly lower dose of nitrates than is used in clinical practice was used in the mentioned study. Although nitrates are used very often, no study has shown a reduction in mortality due to the use of nitrates [84,85,86]. One of the potential limitations of using nitrates is their propensity for tachyphylaxis [87,88].

#### 7.1.2. Natriuretic Peptide Vasodilators

Nesiritide is a recombinant human b-type natriuretic peptide (BNP) whose effects mimic those of endogenous hormones [89]. Several smaller studies have shown the benefit of adding nesiritide to standard therapy for the relief of dyspnea in patients with H-AHF; however, on the other hand, the study by O Conor et al. did not show an effect of the use of nesiritide in reducing dyspnea in patients with H-AHF [90,91,92]. It was not associated with a worsening of renal function, but it was associated with an increase in rates of hypotension. Fu S. et al. explored the efficacy and safety of a modified dosage regimen of nesiritide in patients (≥75 years) with AHF [93]. Nesiritide resulted in improvements in dyspnea and edema and similar adverse effects compared with conventional treatment but did not show a reduction in short-term mortality. The VMAC study showed a statistically significant improvement in dyspnea at three hours postinfusion in patients treated with nesiritide over those treated with NTG or placebo [83]. According to all of the above, the use of nesiritide does not pose a risk to kidney function in AHF; however, its use is associated with the appearance of hypotension, so it should be used with caution.

### 7.2. Diuretics

Although intravenous diuretics are the cornerstone of AHF treatment, especially in patients with fluid overload, they are not the basis of treatment in patients with the H-AHF phenotype [21,88]. Their effect is realized in the increased excretion of salt and water through the kidneys, thus reducing the circulating volume of liquid. As already noted, not all patients with H-AHF are volume-loaded, so routine use of diuretics is not necessary when treating this phenotypic variant. Despite vasodilator therapy, diuretics can be used to control blood pressure in patients with H-AHF [88,94]. However, if patients with H-AHF have volume overload (chronic hypertension leads to activation of the renin–angiotensin–aldosterone system, which leads to fluid retention), then diuretics are used to reduce fluid volume [95]. Furosemide has been used most commonly, but alternatives include bumetanide (1 mg equivalent to 40 mg furosemide) and torsemide (20 mg equivalent to 40 mg). More recent studies such as the ADVOR and CLOROTIC studies favor the addition of acetazolamide and hydrochlorothiazide to standard diuretic therapy, although further studies are needed to prove their real effect on the outcome of patients with AHF [96,97].

### 7.3. ACE Inhibitors

While the use of ACE inhibitors is widely accepted for the treatment of hypertension and chronic HF, the benefit of intravenous ACE inhibitors in patients with AHF has been little studied. To the greatest extent, this is a consequence of the fear of side effects in the direction of hypotension, damage to kidney function and electrolyte imbalance. The main effect of enalaprilat in AHF is reflected in the reduction in arterial blood pressure as well as the effect on splanchnic and arterial circulation [98]. Also, in hypertensive patients with AHF, we often have excessive activation of the renin–angiotensin–aldosterone system, which is why the use of ACE inhibitors could play a significant role [99,100]. The results of the retrospective cohort study by Ayaz et al., which examined the use of bolus IV enalaprilat in hypertensive patients with AHF, showed a good tolerance to the use of enalaprilat and the absence of negative effects on renal function [101].

### 7.4. Serelaxin

Serelaxin is a recombinant form of human relaxin-2, a naturally occurring peptide. By binding to the LGR7 and LGR8 receptors, it activates and enhances the vascular endothelin B receptor and vascular endothelial growth factor (VEGF) and leads to the production of nitric oxide [102]. Its influence on the inhibition of angiotensin II and endothelin was also proven [103]. Serelaxin reduces systemic vascular resistance, increases cardiac output, increases renal blood flow and increases glomerular filtration rate [102,104]. It is administered as a continuous IV infusion. Within the framework of the RELAX-AHF study, the therapeutic effect of serelaxin in the treatment of AHF was examined. Administration of serelaxin led to relief of dyspnea and improvement in other clinical outcomes but had no effect on hospital readmissions. Although no differences were observed in the composite outcome at 60 days, a statistically significant improvement in cardiovascular and all-cause mortality was observed at 180 days [105].

### 7.5. Calcium-Channel Blockers

Although effective antihypertensives, calcium-channel blockers have been poorly studied in AHF. Clevidipine is a rapidly acting, arterial selective intravenous calcium-channel blocker. In the PRONTO pilot study, clevidipine safely and rapidly reduced blood pressure and improved dyspnea [106]. According to the study by Koroki T. and associates, which compared the effectiveness of nicardipine and nitroglycerin in patients with H-AHF, the nicardipine group had a shorter length of hospital stay (17.5 [10.0–33.0] days vs. 9.0 [5.0–15.0] days) than the nitroglycerin group [107].

### 7.6. Urapidil

Urapidil acts as an α1-adrenoceptor antagonist and as a 5-HT1A receptor agonist [108]. According to a study by Yang W. et al., intravenous urapidil compared with nitroglycerin was associated with better blood pressure control and preserved cardiac function [109]. A meta-analysis of seven smaller studies showed similar data [110].

### 7.7. Beta-Blockers

Beta-blockers and other nonvasodilator antihypertensives are currently not indicated in the treatment of H-AHF for acute blood pressure reduction [21,111]. However, there are still conflicting opinions when it comes to the use of beta-blockers in patients with decompensated HF who use these drugs in chronic therapy [112]. Namely, sudden discontinuation of beta-blocker therapy can lead to a rebound phenomenon, i.e., increased sensitivity to beta-adrenergic agonists in patients undergoing long-term therapy with certain beta-blockers after the blocker is abruptly withdrawn [113]. The results of a meta-analysis published by Prins et al. indicate that in patients treated for AHF, both hospital mortality and the rate of rehospitalization are lower when beta-blockers are not excluded from therapy [114].

### 7.8. Respiratory Support

In patients with H-AHF who present with hypoxia, oxygen supplementation is recommended [1]. In the absence of hypoxia, oxygen supplementation should be avoided given the evidence that high concentrations of inhaled oxygen can have adverse hemodynamic effects (decreased cardiac output, increased systemic vascular resistance) in patients with HFrEF [115]. The use of noninvasive positive pressure ventilation (NPPV) is also useful in patients with H-AHF who have significant work of breathing. It was also shown that in pulmonary edema, NPPV can affect a faster reduction in dyspnea and correction of metabolic disorders compared to oxygen therapy alone [116,117]. Vital F.M. et al. showed in a meta-analysis of 32 studies that the use of NPPV leads to a reduction in respiratory distress and the need for intubation in patients with pulmonary edema [118]. Noninvasive positive pressure ventilation should be started as soon as possible in patients with respiratory distress (respiratory rate > 25 breaths/min, SpO_2_ < 90%) [1]. In all patients on NPPV, continuous monitoring of vital functions is necessary with periodic checks of gas exchange. In case of further worsening or respiratory failure, intubation is advised.

Application of inotropic/vasopressor stimulation or short-term circulatory support is mostly reserved for hypotensive patients or when developing cardiogenic shock. In these patients, short-term mechanical surgical support may be necessary to maintain a satisfactory cardiac output and enable adequate organ perfusion. Prophylaxis of deep vein thrombosis is recommended for all hospitalized patients with heart failure, except in case of contraindications for its use [1].

Depending on the precipitating factor that led to heart failure or existing comorbidities, the use of different types of drugs is possible [1]: application of antiarrhythmics in the case of supraventricular and ventricular rhythm disorders, dual antiplatelet therapy and percutaneous coronary intervention in the case of acute coronary syndrome, etc.

## 8. Prognosis of Patients with H-AHF

A study examining patient outcomes during the first hospitalization due to AHF showed that in-hospital mortality was up to 7.5% [119]. The lowest in-hospital mortality in patients with AHF was shown in the ESC-HF Pilot registry (3.8%), while the highest was recorded in the ALARM-HF registry (up to 11%) [14]. Postdischarge mortality up to 3 months was 7% to 11% [13,14,119]. One-year mortality in patients with AHF according to the ESC-HF Pilot registry is 17.4%. On the other hand, within the ADHERE registry, one-year mortality was estimated at as much as 36% [18]. A recent study by Lombardy C. and colleagues showed similar data, where the thirty-day mortality from AHF was about 8%, while the one-year mortality was 20% [12]. Registries have shown that the incidence of rehospitalization ranges between 22 and 30% at 1–3 months and reaches 65% at 1 year of the index AHF hospitalization [120,121]. The importance of hospital readmissions due to AHF is reflected in the fact that each new hospitalization has been shown to correlate with worsening cardiac function, reduced quality of life and a higher incidence of death over a longer follow-up period.

In relation to the geographical distribution, the highest mortality from AHF in the three-month follow-up period is experienced by patients living in South America (17.3%), followed by Western Europe (15.1%), North America (13.3%) and Asia and Pacific (11.6%), and the lowest is in Central Europe (9.3%) [122].

Al-Lawati J.A. et al. showed that low SBP values on admission are an independent predictor of mortality in patients with AHF. The higher the SBP on admission, the better the prognosis of AHF patients, regardless of age or estimated LVEF [45]. The OPTIMIZE-HF investigators reported ~50% of patients with AHF had SBP of >140 mmHg at presentation. According to their study, higher SBP at admission was associated with lower in-hospital mortality rates: 7.2% (<120 mmHg), 3.6% (120–139 mmHg), 2.5% (140–161 mmHg) and 1.7% (>161 mmHg) (*p* < 0.001 for overall difference). Postdischarge mortality rates in the follow-up cohort by SBP at admission were 14.0%, 8.4%, 6.0% and 5.4%, respectively (*p* < 0.001 for overall difference) [123]. Other studies also showed that SBP values are one of the most important predictors of mortality in patients with AHF in intensive care units [22,124].

## 9. Limitations

In this paper, we tried to present the relevant data related to H-AHF in the best possible way. The limitations of this review can be reflected in the fact that the paper used data from the aforementioned literature, which are heterogeneous in terms of the clinical outcomes of the patients, which makes it difficult to generalize and make a clear decision on the application of some therapeutic modalities. Also, it should be borne in mind that it is a retrospective analysis of study results, which, even when conducted with standardized methods, carries a risk of credibility due to the quality and accuracy of the original data entered.

## 10. Conclusions

Acute heart failure caused by arterial hypertension is a specific syndrome that requires urgent diagnosis and treatment. It is defined as a sudden onset of pulmonary congestion in the setting of a systolic blood pressure >140 mmHg and often >160 mmHg. Most patients with H-AHF have previously known heart failure (usually with preserved LVEF), which is why it is necessary to improve diagnostic screening in order to detect both diastolic and systolic dysfunction of the left ventricle. To improve the diagnosis and treatment of H-AHF in general, it is important to educate staff about various precipitating factors that can lead to the worsening of chronic heart failure or the appearance of de novo H-AHF, which is of great importance for both diagnosis and treatment. The diagnosis of H-AHF patients must be quick and precise, both due to the rapid establishment of the diagnosis and due to the adequate selection of the therapeutic regimen. New biomarkers such as soluble suppressor of tumorgenicity 2 (sST2), GDF-15, cystatin C, galectin-3, serum uric acid, microRNAs and low serum chloride, which are predictors of outcomes in AHF, must be used in diagnostics. It is also necessary to monitor the values of markers of renal function (urea, creatinine, proenkephalin), the increase of which is associated with the occurrence of renal weakness and increased mortality. Given that the number of patients with hypertension will increase by 15–20% by 2025, preventive examinations of patients are necessary to diagnose arterial hypertension, which is still often undiagnosed in a large number of patients. According to today’s knowledge, a large number of patients who are treated for arterial hypertension do not reach the target values of arterial blood pressure either because they do not have adequate therapy or because they do not adhere to the therapeutic regimen. In order to better treat arterial hypertension and thereby reduce H-AHF, it is necessary to pay attention to the use of fixed combinations of antihypertensive drugs in order to improve patient adherence. Timely initiation of therapy may be a key factor in the successful treatment of H-AHF, with a positive association between a short time from admission to administration of diuretics and vasodilators resulting in reduced in-hospital mortality. However, it is known that the use of diuretics and vasodilators helps control symptoms in hospital conditions, but their effectiveness in reducing H-AHF recurrence, rehospitalization and distant mortality has not been demonstrated. A possible reason for the poor long-term outcome of these patients is the therapy applied in H-AHF, which was most often tested on patients with HF who had reduced LVEF and were in the phase when they had no episodes of AHF, and not on patients with preserved LVEF, which are most common in H-AHF. Given that the use of diuretics and vasodilators is based on the consensus of opinion of the expert and/or small studies, retrospective studies or registers, a larger number of relevant data on the outcomes and safety of the use of these drugs is necessary. After the discharge of patients treated for AHF, it is recommended to start high-intensity HF therapy and rapid titration of oral therapy with the necessary careful monitoring in the first 6 weeks after discharge in order to reduce the number of rehospitalizations. For now, the main goal is to improve the long-term outcome of these patients. Therefore, further research is needed in the future in the improvement of both diagnostic and therapeutic modalities in order to reduce the mortality of this life-threatening disease.

## Data Availability

Not applicable.
